# A scoping review on two-stage randomized preference trial in the field of mental health and addiction

**DOI:** 10.1186/s12888-023-04676-1

**Published:** 2023-03-23

**Authors:** Sheng Chen, Wei Wang

**Affiliations:** 1 Biostatistics Core, Centre for Addiction and Mental Health, 1001 Queen Street West, Toronto, ON Canada; 2 Center for Complex Interventions, Centre for Addiction and Mental Health, Toronto, ON Canada; 3 College of Public Health, University of South Florida, 13201 Bruce B Downs Blvd, Tampa, FL USA

**Keywords:** Clinical trial, Patient preference, Two-stage randomized preference trial, Mental health, Preference effect

## Abstract

**Background:**

Randomized Controlled Trial is the most rigorous study design to test the efficacy and effectiveness of an intervention. Patient preference may negatively affect patient performance and decrease the generalizability of a trial to clinical population. Patient preference trial have particular implications in the field of mental health and addiction since mental health interventions are generally complex, blinding of intervention is often difficult or impossible, patients may have strong preference, and outcome measures are often subjective patient self-report which may be greatly influenced if patient’s preference did not match with the intervention received.

**Methods:**

In this review, we have surveyed the application of two-stage randomized preference trial with focus on studies in the field of mental health and addiction. The study selection followed the guideline provided by Preferred Reporting Items for Systematic reviews and Meta-Analyses extension for Scoping Reviews.

**Results:**

Six two-stage randomized preference trials (ten publications) have been identified in the field of mental health field and addiction. In these trials, the pooled dropout rates were 18.3% for the preference arm, and 28.7% for the random arm, with a pooled RR of 0.70 (95% CI, 0.56–0.88; *P* = 0.010) indicating lower risk of dropout in the preference arm. The standardized preference effects varied widely from 0.07 to 0.57, and could be as large as the treatment effect in some of the trials.

**Conclusion:**

This scoping review has shown that two-stage randomized preference trials are not as popular as expected in mental health research. The results indicated that two-stage randomized preference trials in mental health would be beneficial in retaining patients to expand the generalizability of the trial.

**Supplementary Information:**

The online version contains supplementary material available at 10.1186/s12888-023-04676-1.

## Background

Randomized Controlled Trial (RCT) is regarded as the gold standard to test the efficacy and effectiveness of a treatment or intervention [1]. When randomization procedure is conducted properly, RCT is the most rigorous study design and can significantly reduce bias such as selection bias among the treatment arms [1]. The limitations of RCT have also been discussed in the literature. The unwillingness of participation may create a gap between the research sample and the targeted population, hence decrease the generalizability of RCT to clinical population [2]. And in trials that interventions are not blinded, when participants are assigned to a non-preferred arm, their performance may be impacted negatively with higher drop-out rate, and/or low adherence to treatment protocol [3].

**Fig. 1:**
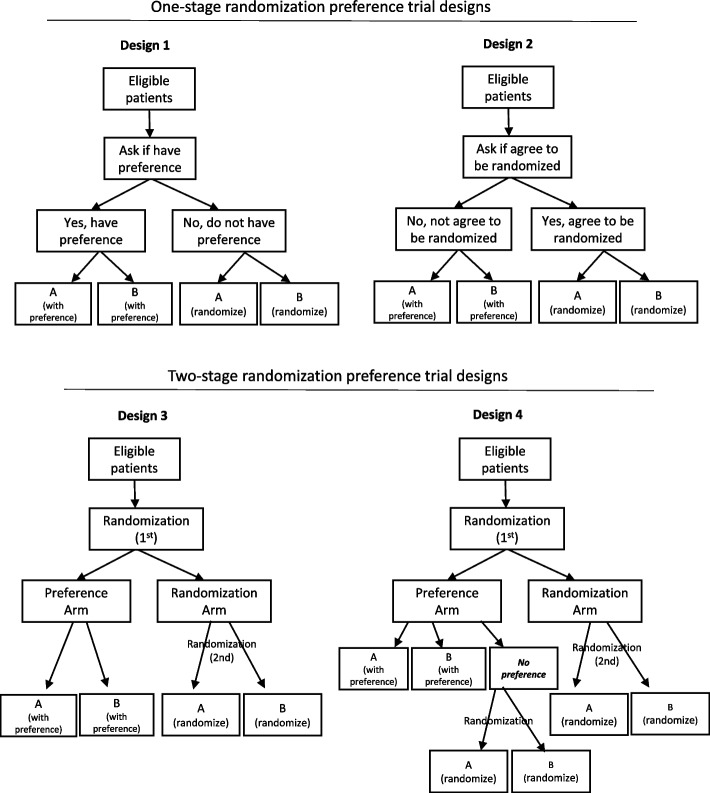
Architectures of patient preference trials

Under this context, the idea of patient preference trial was raised. Kowalski [3] summarized four typical designs of conducting patient preference trials (Fig. 1). In order to evaluate the impact of patients’ preference on treatment effect, these trials contains both by randomization and by preference assignment of treatment condition. In Design 1, patients will be asked if they have preference with the treatments. Patients are allowed to choose their preferred treatment if they have one and will be randomized if they don’t. Design 2 begins with asking patients if they would be willing to be randomized to treatment arms. Those agreeing of randomization are randomly assigned to one of the treatment arms, and those who refusing are offered their preferred treatment. Design 3 starts with randomizing (1^st^ randomization) all eligible and consenting patients to a “preference arm” or “randomization arm”. Patients in the preference arm are offered to choose their preferred treatment, while those in the randomization arm are then randomized (2^nd^ randomization) into one of the treatments. Lastly, Design 4 complements Design 3 by offering randomization to those who are assigned to the preference arm but are unable/unwilling to choose a treatment.

Designs 1 and 2 are called one-stage randomized or partially randomized preference trials, which strictly speaking are observational studies as patients are given options from the beginning. Selection bias are introduced to the design due to patients’ preference thus the outcomes are confounded [4]. Designs 3 and 4 are known as two-stage or doubly randomized preference trials. Since patients are randomly assigned to the preference and randomization arms, the two-stage randomized preference trial designs allow unbiased estimation of treatment effect (the direct effect of treatment), selection effect (the effect of participant’s desired treatment) and preference effect (the interaction between desired and actual treatments) [5], in addition to a greater external validity than the conventional RCT.

Patient preference trial may have particular implications in the field of mental health and addiction [4], since mental health interventions are generally complex, blinding of intervention is often difficult or impossible e.g. medication versus psychological intervention, patients may have strong preference, and outcome measures are often subjective patient self-report which may be greatly influenced if patient’s preference did not match with the intervention received.

There have been systematic reviews on patient preference trials [6–8]. These reviews provided evidence that patient preference increased external validity of the trials. King [6] and Delevry [8] included both one-stage and two-stage randomized trials but it could use an update, and Wasmann [7] focused only on one-stage preference trials. Besides, King [6] and Wasmann [7] did not evaluate the estimation of treatment effect, selection effect and preference effect across the trials. Delevry [8] reported the pooled effect sizes for the preference and choice (selection) effects, but since one-stage randomized trials were included, the estimates are biased and hence questionable. In this review, with up to date literature search, we assess the application of two-stage randomized preference trials with focus on studies in the field of mental health and addiction. The following research questions have been formulated: (a) to what extent the two-stage randomized preference trial has been used in the field of mental health and addiction? (b) does patient preference influence drop-out in two-stage randomized preference trials in the field of mental health and addiction? and (c) what are the pooled preference effect, compared to the treatment effect and selection effect in the same trial, and compared among the trials surveyed by this review?

## Methods

### Eligibility criteria

Peer-reviewed journal papers were included if they were: published from inception to September 2022, written in English, reported a clinical trial in the mental health and addiction field, and with a two-stage randomized patient preference design. Trials published as study protocol only, abstracts, sub-studies and secondary publication of included trials were excluded. For trials reported in multiple publications, we included all the publications since these publication might present different outcome measures, but were considered as one single trial in this review.

### Information sources

To identify potentially relevant publications, the following bibliographic databases have been searched up to September 2022: PubMed, Cochrane Library and Google Scholar. The terms employed included indexing terms (e.g., MeSH) and free texts: patient preference, patient choice, trial, depression, bipolar, schizophrenia, psychosis, dementia, PTSD, OCD, mood disorder, panic disorder, anxiety, autism, ADHD, substance use, drug use, alcohol, nicotine, cannabis, addiction, mental illness, mental disorder, mental health, psychiatry. The syntax used for search in PubMed was: ("participant* choice" OR "participant* preference" OR "client* choice" OR "client* preference" OR "patient* choice" OR "patient* preference") AND (depression OR bipolar OR schizophrenia OR psychosis OR dementia OR PTSD OR OCD OR "mood disorder" OR "panic disorder" OR anxiety OR autism OR ADHD OR "substance use" OR "drug use" OR alcohol OR nicotine OR cannabis OR addiction OR "mental illness" OR "mental disorder" OR "mental health" OR psychiatry).

### Selection of sources of evidence and data extraction

**Fig. 2:**
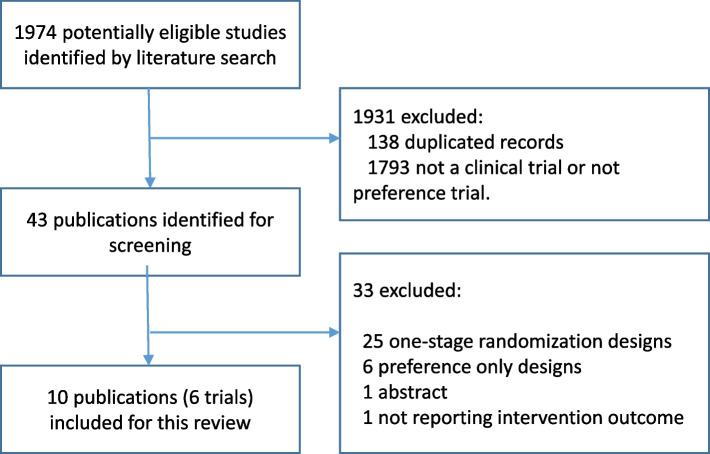
Study selection according to Preferred Reporting Items for Systematic Reviews and Meta-Analysis (PRISMA)

Detailed data of the study selection process are shown in Fig. 2. The two authors separately reviewed the search results for eligible publications. Disagreements were discussed until a consensus was reached.

Data extracted from eligible publications/trials included trial design, sample size, type of treatment, primary outcome measures (key numerical characteristics). Instead of reporting standard deviation for the outcome measures, some publications provided standard error or 95% confidence interval for the means. The standard deviation was converted from standard error or 95% confidence interval [9]. In the cases that the data necessary for the synthesis of result was not available in the publication, effort was made to contact the correspondence authors to request data.

### Synthesis of results

The included studies were summarized by the study population, sample size, interventions, and outcome measures (Table 1). A systematic review was ruled out due to the small number of eligible publications. Accordingly, a scoping review was considered as the more appropriate approach.

**Table 1:** Two-stage randomized patient preference trials included in this review

Trial	Publication	Population	**N**	Intervention and comparison groups	Primary outcome(s)
1	Rokke et al. 1999 [10]	Older adults with depression	35	A: Behavioral therapy B: Cognitive therapy	Depression ratings scales (HDRS, BDI, GDS) immediately, 1 m and 3 m post treatment
2	Hegerl et at. 2010 [11]; Mergl et al. 2011 [12]	Depressed primary-care patients	226	A: Medication (sertraline) B: CBT	Depression ratings scales (HDRS) at 10w
3	He et al. 2018 [13]; Gewirtz et al. 2019 [14]	Parents of children with behavioral problems	128	A: Parent Management Training-Oregon Model (PMTO) B: Services as usual (SAU)	Parenting outcome post intervention (Parental Locus of Control Scale, Family Interaction Task)
					Youth Outcomes post intervention (The Strength and Difficulties Questionnaire)
4	Le et al. 2018 [15]; Zoellner et al. 2019 [16]	PTSD patients	200	A: Medication (sertraline) B: prolonged exposure therapy	Quality of life (EQ-5D) at 10w
					PTSD Symptom Scale–Interview Version [PSS-I] up to 24 m post treatment
5	Brenes et al. 2020 [17]	Older adults with late‐life worry/anxiety	500	A: CBT B: Yoga	Worry scale (PSWQ‐A)(change from baseline tp post treatment)
6	Svensson et al. 2021a [18]; Svensson et al. 2021b [19]	Adults with panic disorder with/without agoraphobia	217	A: Panic Control Treatment B:Panic focused psychodynamic therapy	Panic Disorder Severity Scale (PDSS) post treatment and follow-up 24 m

The sample sizes for each arm and the number of participants who completed the studies were collected from the eligible publications/trials. The dropout rates for the preference arm and randomization arm, and Risk Ratio (RR) of the dropout comparing the preference arm to the randomized arm were calculated for each trial, and the pooled RR was analyzed with R meta and dmetar packages using a random-effects model and the Mantel–Haenszel method.

The treatment effect, selection effect, and preference effect were calculated using the formulae from Walter [5]. Treatment effect is the outcome difference when all participants are randomized to treatment arms (and ignoring the potential complication of noncompliance). The effect of the participant's preferred or desired treatment (if they were allowed to choose) has been termed the selection effect. When comparing two treatments, the selection effect for the two treatments will be opposite to each other. Beyond selection effects, psychological factors such as motivation, engagement, and compliance may be better in patients receive preferred treatments than patients receive a randomized treatment [20]. The preference effect is the effect of whether or not participants actually receive their preferred treatment, which is the additional change in outcome that results from the interaction between a patient’s preferred treatment and the treatment he/she actually receives [5]. Following the same notation used by the authors with a linear decomposition, the expected value at population level of the outcome observed from a participant of treatment assignment $i$ ($i$ =A or B) and preference $j$ ($j$ =A or B) is denoted as

$$E\left({Y}_{ijk}\right)=\mu +{\tau }_{i}+{\nu }_{j}+{\pi }_{ij}$$

Under this notation, the treatment effect, selection effect, and preference effect can be denoted as ${\tau }_{A}-{\tau }_{B}$, ${\nu }_{A}-{\nu }_{B}$, and ${\pi }_{AA}-{\pi }_{AB}-{\pi }_{BA}+{\pi }_{BB}$ respectively.

To estimate these effects with a collected sample from a two-stage design, we need to first calculate the sample means of those assigned to the two conditions within each arm. In the following, we will denote these same means as${\overline{Y} }_{A}$, ${\overline{Y} }_{B}$ (sample means of those in randomization arm) and ${\overline{X} }_{A}$, ${\overline{X} }_{B}$ (sample means of those in preference arm) respectively. Treatment effect, selection effect and preference effect can be estimated using the following formulae:

$$\mathrm{Treatment\;effect}: {\overline{Y} }_{A}-{\overline{Y} }_{B}$$

$$\mathrm{Selection\;effect}: \frac{{m(Z}_{A}-{Z}_{B)}}{2{m}_{A}{ m}_{B}}$$

$$\mathrm{Preference\;effect}:\frac{{m(Z}_{A}+{Z}_{B)}}{2{m}_{A}{ m}_{B}}$$

where ${m}_{A}$ and ${m}_{B}$ are the sample size of the treatments A and B groups in the preference arm, and $m=m_A+m_B$; ${Z}_{A}={m}_{A}({\overline{X} }_{A}-{\overline{Y} }_{A})$; ${Z}_{B}={m}_{B}\left({\overline{X} }_{B}-{\overline{Y} }_{B}\right)$.

To show a direct horizontal comparison of the different effects in the same trial, and a vertical comparison of the effects among different trials, all these effects were calculated both with raw means, and with standardized means (difference of the means with the pooled means and divided by the pooled SD).

## Results

### Selection, inclusion, and characteristics of the trials

A total of 1974 citations were identified from searches of electronic databases and review article references. Based on the title and the abstract, 1931 were excluded, with 43 full text articles to be retrieved and assessed for eligibility. Of these, 33 were excluded for the following reasons: 25 were one-stage randomization preference trials, six were patient preference only observational studies, one was a conference abstract, and one reported only trial retention but not intervention outcome. The remaining 10 publications from six trials were considered eligible for this review [10–19]. All the trials included here adopted the Design 3 two-stage randomized preference trial (Fig. 1).

These six trials covered a few different mental health issues (Table 1). Two of the trials were studies on managing depression [10–12], one targeted on treating PTSD [15, 16], one tested on controlling late-life worry/anxiety in old adults [17], one studied the effect of patient choices on therapeutic outcomes for panic disorder [18, 19], and one assessed the effectiveness of a parent training program for parents whose children had behavioral problems [13, 14].

### Sample size and dropout rate

The sample size ranged widely from 35 to 500 in these six trials (Tables 1 and 2). The dropout rate also varied considerably, from the lowest 4.4% in the preference arm [17] to 75% in the randomization arm [10]. It is evident that preference arm had lower dropout rate than the random arm in all the trials. The RRs comparing the preference arm to the randomized arm ranged from 0.27 to 0.84 for these trials. By pooling the data from these trials, the overall dropout rates were 18.3% for the preference arm, and 28.7% for the random arm (Cluster-weighted Chi-squared test, χ^2^= 2.892, *P* = 0.089). A pooled RR of 0.70 (95% CI, 0.56–0.88; *P* = 0.010) was obtained, implying a lower risk of dropout in the preference arm (Table 2).

**Table 2:** Dropout rates of the preference and randomized arms in the trials included in this review

Trial	Publications	N	Intervention and comparison groups	Dropout rate		RR [95% CI]
				**Preference arm**	**Random arm**	
1	Rokke et al. 1999 [10]	35	A: Behavioral therapy B: Cognitive therapy	20.0%	75.0%	0.27[0.09—0.76]
2	Hegerl et at. 2010 [11]; Mergl et al. 2011 [12]	226	A: Medication (sertraline) B: CBT	30.5%	43.8%	0.70 [0.48—1.01]
3	He et al. 2018 [13]; Gewirtz et al. 2019 [14]	128	A: Parent Management Training-Oregon Model (PMTO) B: Services as usual (SAU)	53.8%	72.3%	0.73 [0.56—0.96]
4	Le et al. 2018 [15]; Zoellner et al. 2019 [16]	200	A: Medication (sertraline) B: Prolonged exposure therapy	28.9%	36.9%	0.78 [0.52—1.17]
5	Brenes et al. 2020 [17]	500	A: CBT B: Yoga	4.4%	8.8%	0.50 [0.28—1.01]
6	Svensson et al. 2021a [18]; Svensson et al. 2021b [19]	217	A: Panic Control Treatment B: Panic focused psychodynamic therapy	10.1%	12.0%	0.84 [0.39—1.79]
Pooled overall (Mantel–Haenszel method,random effects model) *P* = 0.010						0.70 [0.56—0.88]

### Preference effect, treatment effects and selection effect

Due to the unavailability of original data from some of the trials, we were only able to synthesize the treatment effect, selection effect and preference effect for five of the trials (Table 3). To allow readers who are familiar with the outcome measures to get direct information on how large the effects are, and to allow for within-trial comparison among treatment effect, selection effect and preference effect, these effects were first calculated using non-standardized data. As shown in Table 3, in the Brenes [17] trial comparing CBT and Yoga on controlling late-life worry/anxiety, the preference effect (1.54) was almost as big as that of the treatment effect (1.60) on the worry scale outcome measure. The Le [15] trial for treating PTSD with medication or prolong exposure had a preference effect (0.06) that was one third of the treatment effect (0.18) on the patient’s quality of life measure.

**Table 3:** Treatment effect, selection effect and preference effect on the primary outcomes of the eligible trials

Trial	Publication	Treatment and comparison groups	Primary outcome(s)	Means				Treatment Effect (for A)	Selection Effect (for A)	Preference Effect	*Standardized*		
				**Preference Arm**		**Random Arm**					***Treatment Effect*** ***(for A)***	***Selection Effect*** ***(for A)***	***Preference Effect***
				**A**	**B**	**A**	**B**						
1	Rokke et al. 1999 [10]	A: Behavioral therapy B: Cognitive therapy	Depression ratings scales (HDRS, BDI, GDS) immediately, 1 m and 3 m post treatment	Data Not Available				-	-	-	*-*	*-*	*-*
2	Hegerl et at. 2010 [11]	A: CBT B: Medication (sertraline)	Depression ratings scales (HDRS) at 10w ^a^	10.84	9.30	9.32	9.48	-0.16	1.45	0.96	*-0.02*	*0.24*	*0.18*
	Mergl et al. 2011 [12]												
3	He et al. 2018 [13]	A: Parent Management Training-Oregon Model (PMTO) B: Services as usual (SAU)	Parenting outcome (Parental Locus of Control Scale) post intervention ^a^	113.9	119.7	116.8	111.3	-5.47	12.03	1.32	*-0.29*	*0.64*	*0.07*
			Parenting outcome(Family Interaction Task) post intervention	2.43	1.99	2.20	2.37	-0.17	0.77	0.28	*-0.34*	*1.55*	*0.57*
	Gewirtz et al. 2019 [14]		Youth Outcomes (The Strength and Difficulties Questionnaire) post intervention	Data Not Available				-	-	-	*-*	*-*	*-*
4	Le et al. 2018 [15]	A: Prolonged exposure therapy B: Medication (sertraline)	Quality of life (EQ-5D) at 10w	0.83	0.78	0.85	0.67	0.18	-0.12	0.06	*0.79*	*-0.50*	*0.25*
	Zoellner et al. 2019 [16]		PTSD Symptom Scale–Interview Version [PSS-I] up to 24 m post treatment	Data Not Available				-	-	-	*-*	*-*	*-*
5	Brenes et al. 2020	A: CBT B: Yoga	Worry scale (PSWQ‐A) (change from baseline)	9.10	8.40	8.80	7.20	1.60	-0.96	1.54	*0.21*	*-0.13*	*0.21*
6	Svensson et al. 2021a [18] Svensson et al. 2021b [19]	A: Panic Control Treatment B:Panic focused psychodynamic therapy	Panic Disorder Severity Scale (PDSS) post treatment ^a^	8.6	9.7	7.5	10.8	3.30	-2.22	0.23	*0.64*	*-0.43*	*0.04*
			Panic Disorder Severity Scale (PDSS) follow-up at 24 m ^a^	7.1	5.1	6.2	7.5	1.30	-3.53	1.93	*0.24*	*-0.65*	*0.35*

^a^Greater score represents worse outcome

The effects were also calculated with standardized means (Table 3) to facilitate comparisons across trials and between different outcome measures within a trial. Both the Le [15] and Brenes [17] trials had standardized preference effect slightly above 0.2, indicating a small effect size. The He [13] trial had two primary outcome measures on parenting outcomes following treatments. The standardized preference effects varied widely from 0.07 for the Parental Locus of Control Scale to 0.57 for the Family Interaction Task. The primary outcome measure Panic Disorder Severity Scale (PDSS) in Svensson [18, 19] trial was assessed at post treatment and followed up until 24 months post treatment. The standardized preference effect was 0.04 at immediately post treatment, and 0.35 at 24 months post treatment.

## Discussion

In the first stage of the two-stage randomized preference trial, eligible patients are randomly assigned to the preference arm and random arm without selection bias. Patients who are assigned to the preference arm will be allowed to choose treatments based on preference. Patients in the random arm will proceed to the second stage randomization into different treatments, which makes this arm a RCT by itself. Hence the two-stage randomized preference trial design can both keep the rigorousness of the RCT, and expand the generalizability of RCT by possibly allow more patients to participate the trial. In addition, the two-stage randomized preference trial will make it feasible to estimate selection effect and preference effect in an unbiased way [5].

Mental health interventions are often complex with efforts contributed by multidisciplinary members, which makes blinding of treatments very difficult or impossible [4]. Mental health patients usually have strong preference in treatment when choosing between different treatments. For example, in a depression trial [12], of the 145 patients remained in the study, 32 (22.1%) had a preference for anti-depressant medication, and 85 (58.6%) for psychotherapy CBT. In addition, it is common that the outcome measures for mental health trials are from subjective patient self-report [21]. The outcomes may be substantially impacted when patients were assigned to a treatment they do not prefer. Meanwhile, measuring the selection effect and preference effect in mental health clinical trials can be very meaningful for advocating patient-centered or patient-perspective care in mental health and psychiatry practice [22]. Given these advantages, two-stage randomized preference trial could be a good alternative for RCT in mental health research.

However, we were only able to identify six two-stage randomized preference trials (ten publications) in mental health area. A number of reasons could account for this non-popularity. First, the design of the two-stage randomized preference trial is relatively more complex than conventional RCT; second, the two-stage randomized preference trial needs much larger sample size than RCT; and third, in the preference arm, it might be difficult to determine how many patients will choose to enter each intervention, hence the funding agencies may be reluctant to accept estimates of the cost and duration of the trial without results from a pilot study specifically designed to elicit this type of information [4].

With the data available from the six trials reviewed here, the pooled dropout rate is lower in the preference arm than in the random arm with a pooled RR of 0.70 (95% CI, 0.56–0.88; *P* = 0.010). This finding is in line with the conclusions from recent systematic reviews on patient preference trials in general areas [7] and on RCTs in mental health with patient preference data collected prior to randomization [23]. It is apparent that considering patient preference will be beneficial in retaining patients in the study, and may potentially improve patient adherence to protocol.

Psychological factors may contribute to treatment outcomes. Patients who receive preferred treatments generally have better motivation, engagement, and compliance than patients who are randomized to a treatment [20]. Preference effect is the effect of whether or not participants actually receive their preferred treatment, which is an interaction between the desired and actual treatments [5]. Interaction tests generally require relatively large sample size. Some of the earlier trials included in this review seemed to be underpowered with small number of participants, and did not provide necessary data for calculating preference effect. With the data available from five trials, the preference effect varied widely from trial to trial, and showed quite large difference between different outcome measures within the same trial. The diversity of the interventions and target populations, the relative small sample sizes and the high dropout rates of some of the trials, may contribute to the variation of the preference effect. Nevertheless, these trials demonstrated that preference effect is not negligible, and could be as large as or even larger than the treatment effect.

The requirement of larger sample size may have limited the utilization of two-stage randomized preference trial in mental health. Cameron [24] proposed a stratified two-stage randomized patient preference design to allow different preference rates and effect sizes across the entire study population, and achieved greater power with smaller sample sizes. Trialists in mental health may consider this design to increase the efficiency of the trial.

The first step of the two-stage randomized preference trial is to randomize eligible patients into two arms. This step may exclude some patients from participating, since these patients may not be willing to be randomized. Patient’s willingness to be randomized, which is similar to the volunteer effect proposed by Kowalski [3], may also have impact on the treatment outcomes. Future studies could consider modifying the trial design to allow estimation of the effect of randomization willingness.

For this study, we only searched the literature published in English and in the databases/search engines of PubMed, Cochrane Library and Google Scholar. Hence we may have missed some trials published in other languages or collected in other bibliographic databases. We were only able to extract data from five trials to synthesize selection effect and preference effect and these trials had very different population, interventions, and sample sizes. These may limit the generalization of the conclusion of this review.

## Conclusion

This scoping review has shown that two-stage randomized preference trials are not as popular as expected in mental health and addiction research. The data indicated that two-stage randomized preference trials would be beneficial in retaining patients to expand the generalizability of the trial, but the preference effect varied widely across the trials being reviewed.

## Supplementary Information

**Additional file 1: Table S1.** Number of patients dropout in each arm of the two-stage trials included in the review.** Table S2.** Outcome mean and standard deviation in each arm of the two-stage trials included in the review.

## Data Availability

All data generated or analyzed during this study are included in this published article and its supplementary information files.

## Abbreviations

ADHD: Attention-deficit/hyperactivity disorder
CBT: Cognitive behavioral therapy
OCD: Obsessive–compulsive disorder
PDSS: Panic Disorder Severity Scale
PTSD: Post-traumatic stress disorder
RCT: Randomized controlled trial
RR: Risk ratio
SD: Standard deviation
